# Mobile care in the pandemic

**DOI:** 10.2471/BLT.22.020222

**Published:** 2022-02-01

## Abstract

The pandemic is shining a new light on mobile clinics and their operators, prompting discussion about how to optimize their use. Gary Humphrey reports.

Dr Kamil Mohamed Ali knows exactly how far good will can take people. “When I was in Somalia working on polio elimination, we’d come across health workers delivering vaccine in the most remote locations. Even in the middle of the rainy season we’d find them trudging along in the rain carrying a couple of bags, often accessing areas completely cut off by floods when the rains came. I didn’t see how they got their supplies through. I later learned that the NGO (nongovernmental organization) they worked with had staged the supplies when the roads were still viable.”

Making sure health supplies and services get through has since become one of Kamil’s daily concerns. Programme Area Manager for the World Health Organization’s (WHO) Emergency Operations Team, he oversees emergency operations in WHO’s Eastern Mediterranean Region, operations that include deploying 54 mobile clinics in the Syrian Arab Republic.

“The mobile medical teams have proved to be highly effective and often life-saving throughout the Syrian crisis, especially when changing lines of conflict have caused massive displacement of local population and the establishment of temporary settlements and IDP (internally displaced persons) camps,” Kamil says, adding that roughly six million people estimated to be living in such camps would be completely cut off from health-care services without mobile clinics.

This assessment is shared by Dr Muhammad Nahel Ghadri, regional director for Syria at Al Ameen, an international NGO based in Turkey which runs mobile clinics on both sides of the Syrian/Turkish border as well as in other eastern Mediterranean countries. “We not only bring basic primary health and maternity services to the people living in IDP camps, we also refer them to fixed facilities when that is needed,” Ghadri explains.

A familiar sight in conflict zones, mobile clinics have become increasingly visible in less fraught settings during the coronavirus disease 2019 (COVID-19) pandemic. “Mobile clinics are being used to meet a different set of access challenges in the pandemic,” says Dr Andre Griekspoor, a humanitarian interventions expert working in WHO’s Department for Emergency Operations.

“In most countries, the network of fixed facilities has not been affected by COVID, although some facilities have faced disruptions due to staffing or supply chain issues, but, particularly in low- and middle-income countries, there have also been significant demand-side barriers caused by restrictions on population movement and changes in health-seeking behaviour driven by fear of infection or reduced ability to pay.”

“Mobile medical teams [have been] life-saving in the Syrian crisis.”

Kamil Mohamed Ali

Countries across the income range are making use of mobile services to break through some of these barriers, often as part of the adaptations required to ensure safe delivery of essential services, and also to support COVID-19 interventions, including testing, mask distribution and vaccination.

In the Philippines, for example, the Philippine Red Cross has been delivering COVID-19 vaccination using buses that reach populations assessed to be below the poverty line based on data provided by the National Household Targeting System for Poverty Reduction.

The so-called Bakuna Buses are provided by a Manila-based bus company and have customized interiors that allow health workers to administer vaccines inside the vehicle itself. The buses are also equipped with mobile refrigerator units and vaccine cabinets.

“Most of these initiatives are run through nongovernmental or charitable organizations, or supported with philanthropic project-based funding,” comments Griekspoor, noting that they generally fill a niche during an epidemic, or provide very specific services, such as cataract surgery or preventive services as part of community outreach programmes. “The public sector tends not to use them to deliver a broader package of essential services, because, on a pure cost of care per patient basis, they are more expensive than care delivered through fixed facilities,” he adds.

Part of those costs derive from expenditure on maintenance and repairs. “Decades of experience have revealed their weaknesses, notably in terms of reliability,” says Griekspoor. “Put very simply, mobile clinics tend to break down and run out of gas and, depending on the setting in which they are working, that can be an insurmountable problem.”

Balancing the different trade-offs, governments and the health ministries they fund have tended to direct their resources into fixed facilities, creating a space which, for the most part, has been filled by philanthropies/charities, and NGOs.

Al Ameen is one example. Backed by multiple partners, the NGO is heavily dependent on the channelling of good will, most of it coming from Médecins Sans Frontières (MSF), which, according to Francisco Otero y Villar, MSF Head of Mission for northwest Syria, provides funding as well as technical and medical supply support for 15 mobile clinics, including the Al Ameen vehicles. “Some of the mobile clinics are shared between the partners, but most are sponsored directly by MSF,” he says.

Private sector players are also active in the mobile health service delivery, one example being the Projeto CIES initiative in Brazil which parks mobile clinics – a mix of buses, vans and truck-mounted containers – near public hospitals and contracts with city governments or private companies to cover treatment.

Not everyone is convinced that simple cost per patient comparisons capture the full health financing implications of mobile care provision. For example, Sharon Attipoe-Dorcoo, a public health researcher and consultant based in Georgia, United States of America (USA) argues that the cost savings generated by effective prevention should be factored in. “There may be an upfront cost in supporting mobile clinics, but by bringing preventive services into communities that would not otherwise access them, they generate tremendous savings down the line,” she says.

Lead author of an article published in the December 2020 issue of the International Journal for Equity in Health (IJEH) which highlights opportunities for policy change and innovation with regard to mobile health clinics that have emerged during the pandemic, Attipoe-Dorcoo cites as an example Harvard Medical School's Family Van, an inner-city project, that brings screening and referral services to some of Boston’s underserved neighbourhoods. These include screenings for blood pressure, cholesterol, blood glucose, obesity, depression, vision and glaucoma as well as pregnancy testing, family planning services, and HIV counselling.

“Mobile clinics are being used to meet [different] access challenges in the pandemic.”

Andre Griekspoor

According to the IJEH paper, some 2000 mobile clinics operate across the USA, serving around seven million people annually. Many have played key service delivery roles during the pandemic, including the delivery of COVID-19-related services. One example is the seven mobile clinics working with the Parkland Health and Hospital System in Dallas, which have served as either drive-through COVID-19 testing sites or triage locations in the parking lots near the emergency department or at regional vaccination delivery sites.

For Griekspoor, cost comparisons and other implementation questions aside, there is an argument for greater engagement by government with the plethora of actors providing health services with mobile clinics, particularly in the context of humanitarian crisis response.

“An NGO-run mobile team may be an off-budget investment from the point of view of a health or finance ministry, but it is an investment in health services, nevertheless. Policy-makers have a key role to play in making sure that investment is optimized, and that these services become connected with the broader health network for referral, and its governance and oversight by district health authorities.”

A first step in harnessing the good will of actors ready to invest is to make sure that it is all pulling in helpful directions and preferably in collaboration. On the face of it this would seem like a difficult task, but it has been done, notably in Syria and with WHO’s support.

Encouraging collaboration and ensuring quality of care were among WHO’s main goals in developing the Syrian Arab Republic Health NGOs Strategy for Engagement in The Health Sector 2017–2022. The strategy was drawn up with the participation of 40 national NGOs working in the health sector and has served as a framework for WHO and other United Nations agencies’ engagement with non-state health actors in the country.

“A core aim of the strategy was to initiate an essential basic health service package required of every provider, as well as the norms and standards needed to engage in service delivery in terms of infrastructure, equipment, medicines, supplies as well as the human resources,” Kamil explains.

The strategy also provided a framework for training needs assessment to develop a comprehensive coherent human resource development programme for NGOs and a framework for harmonizing the work of NGOs as a network to consolidate their contribution to the health sector.

“We are committed to providing all the technical and operational support to NGOs and local communities in contributing to the resilience of the country health system,” says Kamil. “And despite all the challenges faced, that effort has been largely successful so far, with NGOs ensuring access in even the hardest to reach areas, supporting fixed facilities where possible, and mobile clinics where needed.”

**Figure:**
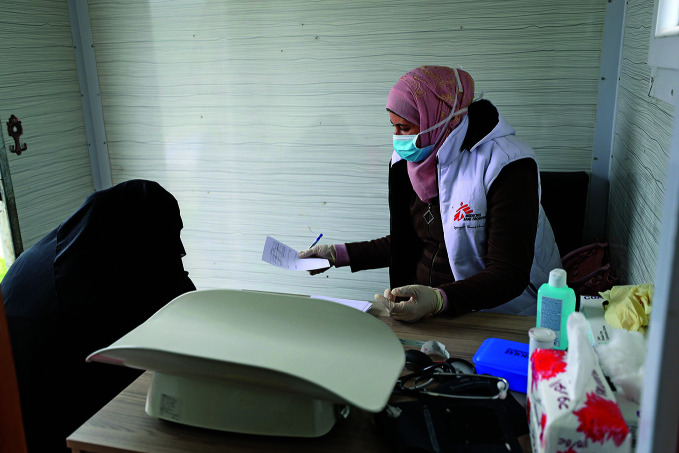
A consultation inside an MSF mobile clinic in a Syrian IDP camp.

Omar Haj Kadour/MSF

**Figure:**
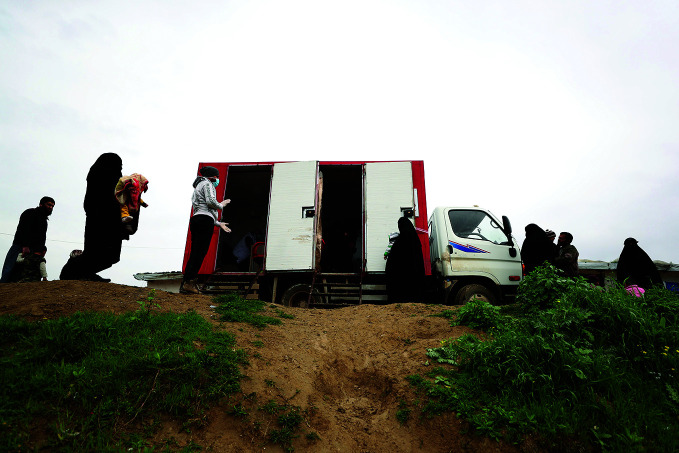
People observe social distancing measures outside an MSF mobile clinic in a Syrian IDP camp.

Omar Haj Kadour/MSF

